# *Pandanus Amaryllifolius Roxb*. Polyphenol Extract Alleviates NAFLD via Regulating Gut Microbiota and AMPK/AKT/mTOR Signaling Pathway

**DOI:** 10.3390/foods14061000

**Published:** 2025-03-15

**Authors:** Jinji Lin, Fei Ren, Mengxu Zhu, Yibo Hu, Zhiao Zhao, Jianfei Pei, Haiming Chen, Weijun Chen, Qiuping Zhong, Ying Lyu, Rongrong He, Wenxue Chen

**Affiliations:** College of Food Sciences & Engineering, Hainan University, 58 People Road, Haikou 570228, China; lin054940@163.com (J.L.); renfei0006@163.com (F.R.); zhumengxu8930@163.con (M.Z.); hyb23hndx@163.com (Y.H.); zzahbxy@163.com (Z.Z.); peijianfei@hainanu.edu.cn (J.P.); hmchen168@126.com (H.C.); chenwj@hainan.edu.cn (W.C.); hainufood88@163.com (Q.Z.); lyuying@hainanu.edu.cn (Y.L.)

**Keywords:** *Pandanus amaryllifolius Roxb*. polyphenol extract, NAFLD, lipid metabolism, AMPK/AKT/mTOR signaling pathway, gut microbiota

## Abstract

With the drastic changes in lifestyle, nonalcoholic fatty liver disease (NAFLD) has become a widespread health problem. Natural actives such as polyphenols have multi-target, multi-mechanism characteristics, and offer new opportunities for NAFLD treatment. This study established a high-fat diet (HFD)-induced NAFLD model in mice to investigate the molecular mechanism of *Pandanus amaryllifolius Roxb.* polyphenol extract (PAE) in alleviating NAFLD. The results showed that PAE significantly inhibited HFD-induced obesity, maintained glucose homeostasis, mitigated oxidative damage in liver tissue, and reduced liver steatosis. Moreover, PAE treatment remarkably reversed 16 endogenous DMs, and significantly affected glycerophospholipid metabolism, which increased the levels of phosphatidylcholine and phosphatidylethanolamine, and down-regulated choline and sn-glyceropl-3P. Further validation revealed that PAE was able to prevent NAFLD progression by regulating the AMPK/AKT/mTOR signaling pathway to enhance autophagy levels. Meanwhile, PAE treatment restored the balance of gut microbiota mainly by increasing the relative abundance of *Bacteroidetes*, decreasing the relative abundance of *Firmicutes* and the ratio of *Firmicutes*/*Bacteroidetes*. Overall, the findings highlight that the mechanism by which PAE alleviates NAFLD may be related to the regulation of the gut microbes and AMPK/AKT/mTOR signaling pathway, enriching the health-promoting effects of PAE on NAFLD.

## 1. Introduction

Nonalcoholic fatty liver disease (NAFLD) is a chronic metabolic syndrome characterized by hepatic steatosis and metabolic disorders [1]. With the drastic changes in lifestyle, the prevalence of NAFLD has exceeded 25%, posing considerable challenges to healthcare systems. Furthermore, NAFLD is strongly related to multiple metabolic and insulin resistance diseases, including cardiovascular disease, T2DM, and chronic kidney disease [2]. Currently, NAFLD can only be controlled by following a scientifically sound dietary regimen, correcting poor eating habits, engaging in moderate aerobic exercise, and undergoing regular physical examinations. Particularly, therapies for advanced liver diseases are limited, often accompanied by serious side effects. Moreover, there are currently no FDA-approved pharmacological treatments specifically for NAFLD. Therefore, it is imperative to seek safe natural active ingredients to prevent and mitigate the development of NAFLD.

There are many natural actives like flavonoids, alkaloids, polysaccharides, and polyphenols that can be used to prevent NAFLD. There was evidence that dietary antioxidants, particularly polyphenolic compounds, significantly influence NAFLD and its progression [3]. The regular intake of polyphenols can be effective in reducing the risk of various metabolic diseases, including NAFLD [4,5]. It has been reported that phytic acid can ameliorate HFD-induced NAFLD by preserving the integrity of the intestinal barrier, modulating the diversity and composition of intestinal microbiota, and improving the balance of the gut–hepatic axis [6]. Similarly, blueberry leaf polyphenols have been shown to alleviate NAFLD, primarily by improving mitochondrial dysfunction, and reducing liver steatosis, oxidative stress, and inflammation [7]. These findings underscore that polyphenols and natural ingredients rich in these compounds possess positive effects on the prevention and treatment of NAFLD.

*Pandanus amaryllifolius Roxb.* (*P. amaryllifolius*) is a tropical plant, mainly distributed in Hainan, India, South China, and Southeast Asia. It is often propagated and cultivated as a spice due to its medicinal properties including antiviral, antioxidant, anticancer, clotting, and antibacterial activities [8]. *P. amaryllifolius* can not only be made into a variety of special food but also be used as a raw material for spice additives for food, daily necessities, and cosmetics. It is worth mentioning that *P. amaryllifolius* is also rich in phenolic components, flavonoids, alkaloids, β-sitosterol, carotenoids, and other bioactive substances, which show potential application value in the health food industry [9]. The study has confirmed that phenolic-rich *P. amaryllifolius* can protect the liver of male rats from damage and alleviate acute liver damage caused by carbon tetrachloride (CCl4) [10]. However, the hepatoprotective impact of *P. amaryllifolius* phenolic extract (PAE) has not been sufficiently appreciated, especially its mitigation effects on NAFLD, and the underlying mechanisms remain unclear.

It is believed that the imbalance of the gut microbiota often leads to the occurrence of NAFLD, especially the imbalance between *Firmicutes* and *Bacteroidetes* [11]. In recent years, the action of the enterohepatic axis in NAFLD has been gradually exposed due to the elaborate connection between the gut and liver. A high-fat diet (HFD) not only raises the levels of free fatty acids but also alters the diversity and composition of the gut microbiota. Additionally, dysbiosis induced by HFD can lead to chronic intestinal inflammation and impairment of the gastrointestinal barrier, which in turn fosters insulin resistance [12]. This suggested that HFD exacerbated liver steatosis by impacting the balance of the gut–liver axis. Supplementation with noni fruit phenolic extracts has been reported to restore gut microbiota dysbiosis caused by HFD, thereby alleviating NAFLD [13]. Similarly, caffeic acid exerted a protective function against the occurrence of NAFLD by restoring the imbalance of gut microbiota [14]. Therefore, maintaining the balance of the gut–liver axis has emerged as a potentially viable concept to ameliorate NAFLD.

Based on this, this study aimed to investigate the preventive impact of PAE on HFD-induced NAFLD in mice and its potential mechanisms by detecting liver metabolic profile and alterations in gut microbiota. These findings help to reveal the mechanism of PAE in the treatment of NAFLD and provide convincing evidence for the exploitation of *P. amaryllifolius.*

## 2. Materials and Methods

### 2.1. Materials

The *P. amaryllifolius* was obtained from Xinglong Tropical Botanical Garden (Haikou, China), where good moisture regimes are present in air and soil, and no plant growth regulators were used in its cultivation. Healthy and green leaf samples were collected from the site, with no signs of insect or microbial damage in June. Simvastatin was acquired from Renhe Pharmacy Co., Ltd. (Jiujiang, China). AB-8 macroporous resin and Folin-Ciocalteu were obtained by Shanghai Yuanye Bio-Technology Co., Ltd. (Shanghai, China). The triglycerides (TGs), catalase (CAT), total cholesterol (TC), low-density lipoprotein cholesterol (LDL-C), aspartate aminotransferase (AST), high-density lipoprotein cholesterol (HDL-C), superoxide dismutase (SOD), Malondialdehyde (MDA), and alanine transaminase (ALT) were assayed by the appropriate kit (Suzhou Grace, Suzhou, China).

### 2.2. Preparation of PAE

The preparation of PAE was based on previous study [15] and briefly, the *P. amaryllifolius* leaf powder was blended with 70% ethanol (1:10, *w*/*v*) and extracted using ultrasonic extraction (240 W, 40 °C, 30 min). The resulting mixture was centrifuged (8000 rpm, 5 min, 4 °C) and this process was repeated twice to obtain the supernatant. The supernatant was then mixed with an equal amount of ethanol and left overnight at 4 °C to facilitate the removal of carbohydrates and proteins. The supernatant obtained was concentrated in rotary evaporation at 45 °C and then purified by a column filled with AB-8 macroporous resin. After that, non-phenolic compounds were removed with distilled water and adsorbed polyphenolics were eluted with 95% ethanol. The polyphenol content was determined by the Folin–Ciocalteu method [16]. Finally, the polyphenol-containing eluate was subjected to rotary evaporation and freeze-drying to obtain PAE.

### 2.3. Determination of the Composition of PAE

The composition of PAE was analyzed using ultra-performance liquid chromatography–tandem mass spectrometry (UPLC-MS/MS). The ACQUITY UPLC HSS T3 column (2.1 × 100 mm, 1.8 µm) from Waters (Milford, MA, USA) was utilized. The mobile phase A consisted of water with 0.1% formic acid while mobile phase B was acetonitrile with 0.1% formic. The elution procedure was performed as previously described [17]. Mass spectrometric detection of metabolites was conducted using Orbitrap Exploris 120 (Thermo Fisher Scientific, Waltham, MA, USA) equipped with an ESI ion source. The parameters were as follows: sheath gas pressure, 40 arb; aux gas flow, 10 arb; spray voltage, 3.50 kV and −2.50 kV for ESI(+) and ESI(−), respectively; capillary temperature, 325 °C; MS1 range, *m*/*z* 100–1000; MS1 resolving power, 60,000 FWHM; number of data-related scans per cycle, 4; MS/MS resolving power, 15,000 FWHM; normalized collision energy, 30%; dynamic exclusion time, automatic. Analysis of raw data and comparison with databases to enable qualitative analysis of PAE.

### 2.4. Animals and Experimental Design

**Figure 1:**
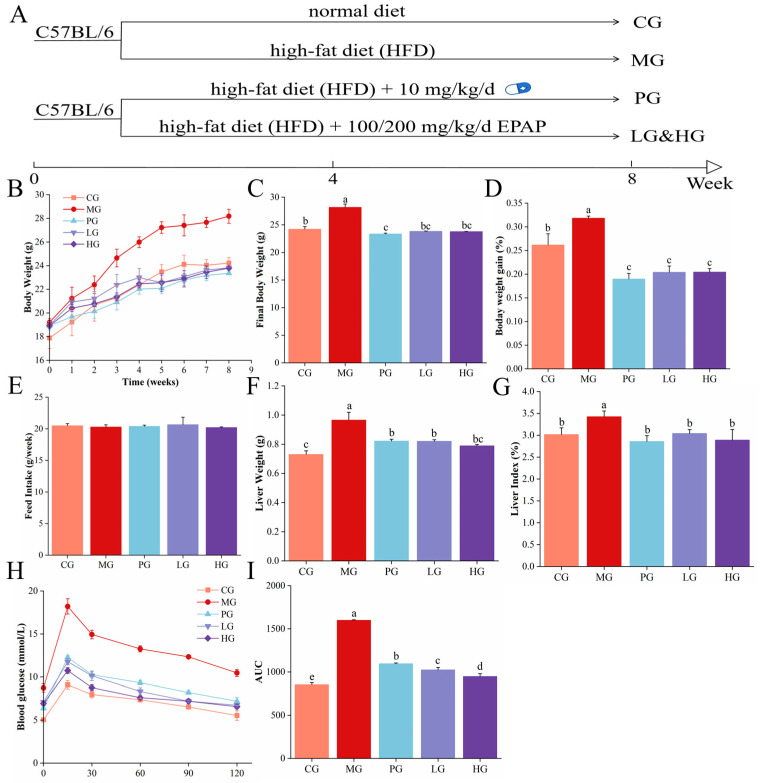
PAE prevents NAFLD in HFD mice. (**A**) Flowchart for establishing C57BL/6 mouse model. (**B**) Body weight. (**C**) Final body weight. (**D**) Body weight gain. (**E**) Feed intake. (**F**) Liver weight. (**G**) Liver index. (**H**) Blood glucose change. (**I**) Area under the curve (AUC). Different letters indicate significant differences between groups (*p* < 0.05).

Forty male C57BL/6 mice (5 weeks) were provided by Slac Jingda Laboratory Animal Co., Ltd. (Hunan, China). The research scheme was approved by Hainan University Animal ethics committee (approval number HNUAEC23-0526). After acclimatization feeding, the mice were classified randomly into five groups (n = 8), as shown in Figure 1A: the control group (CG), the model group (MG), the positive control group (PG, 10 mg/kg, the simvastatin), the low-dose PAE group (LG, 100 mg/kg), and the high-dose PAE group (HG, 200 mg/kg). Mice in MG, PG, LG, and HG were fed HFD daily to establish an NAFLD mouse model. Animal experiments were conducted for 8 weeks. The formulas for the visceral index and relative weight gain rate refer to previous studies [18].

### 2.5. Oral Glucose Tolerance Test (OGTT)

The experiments were performed based on previous methods [19]. In short, mice were gavaged with a glucose solution, and then blood glucose levels were assessed at 0, 15, 30, 60, 90, and 120 min.

### 2.6. Serum and Liver Biochemical Analysis

The serum levels of TC, TG, AST, LDL-C, MDA, HDL-C, SOD, MDA, GSH, and ALT were assayed by the appropriate kit. Liver samples were homogenized in an ice bath with ethanol and then centrifuged (12,000× *g*, 4 °C, 15 min) to gain supernatant. The levels of MDA, SOD, and CAT in liver were also determined using appropriate kits, as described in the manual.

### 2.7. Histological Analysis

Paraformaldehyde-fixed livers were embedded in paraffin, then cut into 4 µm sections and stained with hematoxylin and eosin (H&E) or Oil red O (ORO). Finally, the obtained sections were visualized using a microscope.

### 2.8. Untargeted Metabolomics of Liver

UPLC-MS/MS platform was used to analyze metabolites of liver. Liver samples were eluted on a T3 column (Waters ACQUITY Premier HSS T3 column 1.8 µm, 2.1 mm × 100 mm). The elution procedure was performed as previously reported [20]. Mass spectrometric detection involved the use of AB SCIEX Triple TOF 6600 (Foster City, CA, USA) with an ESI ion source. Data were preprocessed with ProteoWizard, and analyses were performed using R 4.3.2, KEGG (http://www.genome.jp/kegg/), and MetaboAnalyst5.0 (http://www.metaboanalyst.ca/).

### 2.9. Western Blot Analysis

WB was executed as previously measured [21]. Briefly, the lysate was added to the liver tissue and the mixture was homogenized using an automatic grinder. Subsequently, the sample was lysed on ice to obtain total protein. The protein concentration was tested through the BCA protein assay kit. Denatured protein samples were loaded onto a pre-prepared SDS-PAGE gel to obtain the stripes. The stripes were incubated with the antibody. Finally, relative protein expression was assessed by immersing the membrane in ECL luminescent solution, and the data were analyzed using ImageJ 1.8.0

### 2.10. 16 S rRNA Analysis of Gut Microbiota

Microbial diversity was measured as previously described [22]. Briefly, fresh fecal samples were collected before the end of the experiment and immediately placed in liquid nitrogen. A™ Mag-Bind Soil DNA Kit (Omega Bio-Tek, Norcross, GA, USA). DNA concentration and purity were determined on 1% agarose gels electrophoresis and Qubit analysis. For amplification, the variable regions V3-V4 of the 16S rRNA gene were targeted using the 338F/806R primers. To prepare the sequencing libraries, the TruSeq^®^ DNA PCR-Free Sample Preparation Kit (Illumina, San Diego, CA, USA) was utilized. Finally, the libraries were sequenced on the Illumina NovaSeq platform, and analyzed using an online platform.

### 2.11. Statistical Analysis

Data were presented as the mean ± standard deviation (SD). One-way analysis of variance (ANOVA) and Duncan’s test were performed on the biochemical indices using SPSS 26.0, and *p* < 0.05 was regarded as significant differences.

## 3. Results and Discussion

### 3.1. Compounds of PAE

The PAE was characterized by UPLC-MS/MS, and the top 20 polyphenolic compounds are shown in Table 1, which were mainly flavonoids and phenolic acids. These ingredients have recognized biological activities including lipid regulation, anti-inflammatory, blood sugar, and antioxidant. Research indicated that quercetin and kaempferol derived from Carthamus tinctorius L. have the potential to treat NAFLD by modulating NR1H4-mediated pathways [23]. In addition, ferulic acid, caffeic acid, etc., also have a wide range of applications in anti-aging, anti-diabetic, anti-hyperlipidemia, and treatment of NAFLD [24,25]. These studies inspired us to investigate the impact of PAE on NAFLD induced by HFD.

### 3.2. PAE Improved Body Weight Parameters in NAFLD Mice

The effects of PAE administration on the body weight parameters of HFD-induced NAFLD mice are illustrated in Figure 1B–G. As a whole, the body weights of all groups increased steadily during the first month while the mice body weight of the PG, LG, and HG showed a decrease in the 4th week, followed by a decline in weight gain (Figure 1B). Mice in the MG were the heaviest (Figure 1C), with a growth rate of nearly 32%, finally, whereas the PG, LG, and HG have significantly lower body weight growth rates (Figure 1D, *p* < 0.05). Importantly, feed intake did not differ significantly among the groups (Figure 1E). Compared with CG, both the liver weight and index in MG were significantly elevated, as presented in Figure 1F,G (*p* < 0.05). However, the liver weight and index were significantly lower in the PG, LG, and HG than those in the MG (*p* < 0.05). These findings align with a previous study, in which quercetin and its glycoside derivatives effectively reduce the body weight, liver weight, and liver–weight ratios resulting from an HFD [26]. Moreover, there is growing evidence that polyphenol-rich extracts can alleviate HFD-induced body weight abnormalities and liver dysfunction in mice [27]. Thus, the present data reveal that PAE reverses the negative effects of HFD-induced NAFLD and offers significant hepatoprotective benefits.

### 3.3. PAE Maintained Glucose Homeostasis

Fasting blood glucose and glucose tolerance were assessed to evaluate the impact of PAE intervention on glucose tolerance in NAFLD mice (Figure 1H,I). After 12 h of fasting, the MG has the highest fasting blood glucose (8.73 ± 0.53 mmol/L), significantly exceeding the CG (5.03 ± 0.07). After intragastric glucose administration, blood glucose levels in all groups showed an increasing trend in the first 15 min. Between 15 and 30 min, these levels decreased sharply before stabilizing. The MG has the strongest response to glucose intake, and the blood glucose value increased fastest to 18.2 ± 1.00 mmol/L. In addition, the area under the glucose curve (AUC) was significantly greater in the MG than CG, whereas the AUC was significantly lower in the PG, LG, and HG compared to the MG (Figure 1I, *p* < 0.05), revealing that simvastatin and PAE intervention improved glucose tolerance in NAFLD mice. Insulin resistance, named “first hit”, is not only one of the “multiple strikes” that is a key contributor to the onset of NAFLD and its progression to NASH, but also a key factor in activating the lipotoxicity, oxidative stress, and inflammatory cascades [28]. Worse, insulin-resistant adipose tissue can produce excessive free fatty acids through lipolysis, creating a detrimental cycle of metabolite accumulation, steatosis, and further insulin resistance [29]. At the same time, many studies have emphasized the beneficial role of polyphenols in this regard. The α-glucosidase enzyme located on the brush border surface of intestinal cells hydrolyse oligosaccharides to glucose, which is then transported through the intestinal epithelium into the bloodstream, contributing to the rise in blood glucose [30]. However, polyphenols have the ability to inhibit glucosidase activity, which is important for lowering postprandial blood glucose levels. This observation aligns with our findings, which demonstrate that PAE effectively regulates blood glucose levels and aids the maintenance of glucose homeostasis.

### 3.4. Pathological Observations of Liver

**Figure 2:**
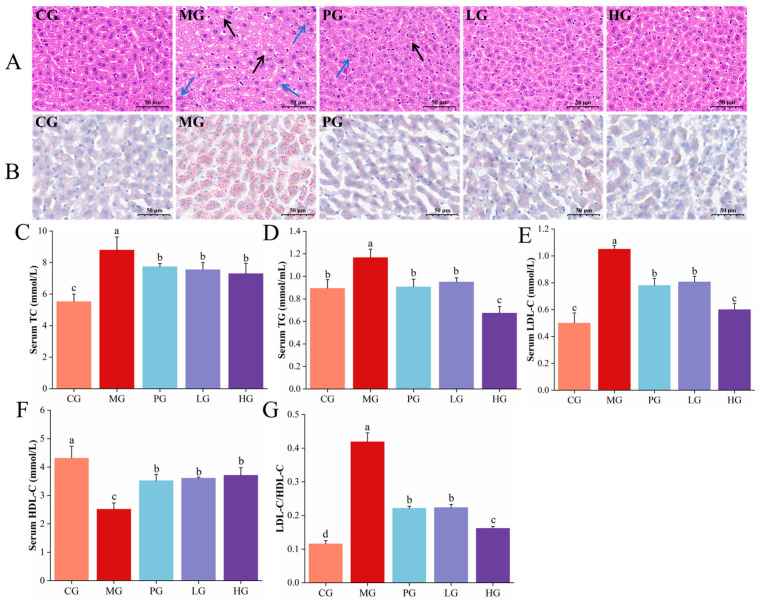
PAE alleviates liver injury in HFD mice. (**A**) H&E staining of mice liver section. (**B**) ORO staining of mice liver section. (**C**–**G**) TC, TG, LDL-C, HDL-C, and LDL-C/HDL-C levels in serum. The black arrow is a fat vacuole, and the blue arrow is an inflammatory cell infiltration. Different letters indicate significant differences between groups (*p* < 0.05).

In this study, H&E and ORO staining were employed to examine liver pathology sections, specifically to observe the effects of PAE intervention on fat vacuoles and inflammatory cell infiltration. As illustrated in Figure 2A, the result of H&E staining showed that a large number of fat vacuoles could be seen in the hepatocytes of MG. Hepatocyte morphology was protected and a marked reduction in fat vacuoles in LG and HG, indicating decreased hepatic lipid accumulation. As presented in Figure 2B, the distribution of lipids and nuclei was clearly visualized in the liver cells of mice after ORO staining. Similarly, lipid droplet staining red was denser in the MG compared to CG, while the volume and density of fat droplets were differentially regulated across PG, LG, and HG. Hepatic lipid deposition is a characteristic pathological hallmark of NAFLD and serves as a primary diagnostic indicator of the disease [31]. There has been a study confirming the ability of polyphenols from plants to alleviate fat accumulation in the liver, among which nobiletin has been found to have the potential to ameliorate liver lobular structural disorder, steatosis, inflammatory infiltration, and fibrosis resulting from methionine and choline-deficient diets in mice [32], consistent with our findings. Overall, PAE intervention can attenuate HFD-induced NFALD by alleviating hepatic fat accumulation.

### 3.5. PAE Improved HFD-Induced Lipid Accumulation

The progression of NAFLD is often linked to metabolic syndromes such as hyperlipidemia, which is mainly characterized by abnormally elevated lipid levels [33]. In this study, biochemical indicators related to blood lipids including TG, TC, HDL-C, and LDL-C were tested to assess the effect of PAE on lipid accumulation in NAFLD mice. The TC and TG of MG were substantially higher compared with the CG (Figure 2C,D). After PAE intervention, the hyperlipidemia of LG and HG decreased to varying degrees (*p* < 0.05), which was in agreement with the results observed in the liver pathology sections. As an important indicator of the body’s lipid metabolism level, once blood levels of TG and TG rise above normal, they can accumulate in blood vessels, leading to clot formation, thereby contributing to the development of chronic metabolic diseases [34]. Likewise, serum LDL-C and LDL/HDL were reduced by 23.17% and 46.76% in the LG, respectively, and HDL-C levels were elevated by 43.12% compared with the MG group (Figure 2E–G). In the HG, serum LDL-C and LDL/HDL were decreased by 42.86 and 61.48%, respectively, and HDL-C content was increased by 47.22%. High density lipoprotein belongs to the body’s beneficial factors and can work in concert with phospholipids to carry cholesterol from circumventive tissue to liver on digestion, which is considered an important indicator of the recovery of cardiovascular and cerebrovascular diseases [35]. In contrast, low density lipoprotein is the primary carrier of cholesterol transported from the liver to other tissues, and there is a strong correlation between LDL-C levels and the occurrence of obesity [36]. Current data suggested that PAE can alleviate HFD-induced abnormal elevation of lipid levels by lowering TC, TG, and LDL-C levels while enhancing the HDL-C level, which was also supported by a similar study [37].

### 3.6. PAE Ameliorated HFD-Induced Liver Damage in NAFLD Mice

**Figure 3:**
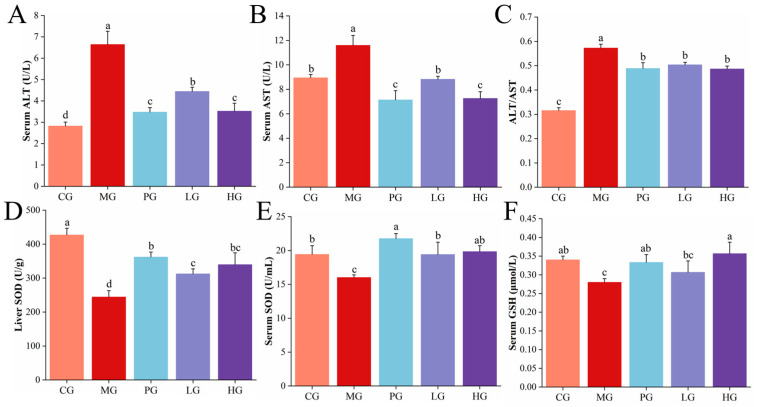
Effects of PAE on blood and liver composition in HFD mice. (**A**–**C**) ALT, AST, and ALT/AST levels in serum. (**D**) SOD level in liver. (**E**,**F**) SOD and GSH levels in serum. Different letters indicate significant differences between groups (*p* < 0.05).

Excessive lipid accumulation will affect the permeability of the hepatocyte membrane and lead to the release of glutamine and ghrelin from the liver into the bloodstream, resulting in an increase of serum aminotransferase levels [38]. Therefore, blood levels of ALT and AST serve as sensitive indicators of liver cell damage. As illustrated in Figure 3A,B, the serum AST was about 11.59 U/L and ALT was about 6.64 U/L in the MG of mice, which were much higher than those of CG (*p* < 0.05). Following low-dose PAE intervention, AST and ALT were much reduced (*p* < 0.05), which were almost recovered to normal levels. The levels of AST decreased to 7.25 U/L and ALT decreased to 3.52 U/L after high-dose PAE intervention. Meanwhile, significantly lower ALT/AST levels were observed in the PG, LG, and HG contrast with the MG (Figure 3C, *p* < 0.05). Based on the present findings, elevated ALT and AST indicated the onset of NAFLD, while PAE treatment reversed serum ALT and AST levels, demonstrating a protective effect against liver injury.

### 3.7. PAE Alleviated HFD-Induced Oxidative Damage of NAFLD Mice

The “second hit” is considered to be the irritation response and cell death, resulting from oxidative stress and endotoxins, ultimately leading to NASH and fibrosis [39]. Therefore, the occurrence of NAFLD may disrupt the balance between antioxidant and oxidative states, and we assessed the impact of PAE on oxidative stress in mice with NAFLD by determining the activities of SOD and GSH. As shown in Figure 3D, compared with CG, SOD activity was markedly decreased by 42.75% (*p* < 0.05) in MG of liver. After simvastatin intervention, SOD was significantly higher (*p* < 0.05). SOD activity was significantly elevated by 27.83% after low-dose PAE intervention. After high-dose PAE intervention, SOD activity was significantly elevated by 38.97%. The serum test results corresponded with those of the liver, revealing that the activities of SOD and GSH of MG showed significant decreases (Figure 3E,F, *p* < 0.05), contrasted with CG. After PAE intervention, the activity of SOD and GSH were restored to varying degrees (*p* < 0.05). A previous report has found that GSH and SOD activities were all reduced in patients with steatohepatitis [40]. Increased antioxidant enzyme activity effectively protects the body from oxidative stress, especially polyphenols, such as phenolic acids and flavonoids, which show significant antioxidant capacity against oxidative stress. Plant polyphenols from grape residues, fruits, and green teas have been shown to increase the activity of antioxidant protective enzymes in different animal models, such as rats [41] and rabbits [42]. Our study also revealed that PAE intervention can alleviate lipid peroxidation by enhancing hepatic antioxidant enzyme activities in NAFLD mice, in turn attenuating liver injury.

### 3.8. Liver Metabolomics Analysis

**Figure 4:**
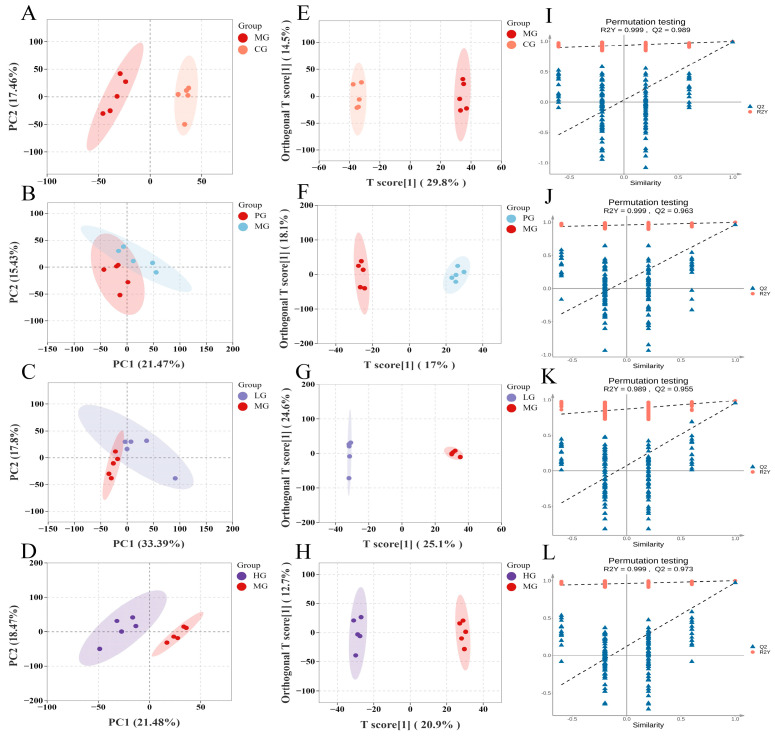
Effects of PAE on liver metabolites. (**A**–**D**) PCA analysis. (**E**–**H**) OPLS-DA analysis. (**I**–**L**) The permutation test results of the OPLS-DA model.

As an important organ of metabolism, the liver plays a crucial role in nutrient transport, immune response, and detoxification within the body [5]. Therefore, we utilized UPLC-MS/MS to identify metabolites in mice liver samples to better understand the material basis of PAE’s ability to mitigate NAFLD and the network pathways involved. A total of 5295 metabolites were detected, with 3041 and 2254 identified in the positive and negative ion mode. The identified metabolites were then subjected to bioinformatics analyses, including clustering through principal component analysis (PCA) and orthogonal partial least squares discrimination analysis (OPLS-DA) plots (Figure 4). The PCA results revealed a complete differentiation between the CG and MG, partially overlapped in the PG, LG, and MG, with completely differentiation between the HG and MG, suggesting that the high-dose PAE intervention reversed the metabolite alterations induced by HFD feeding.

**Figure 5:**
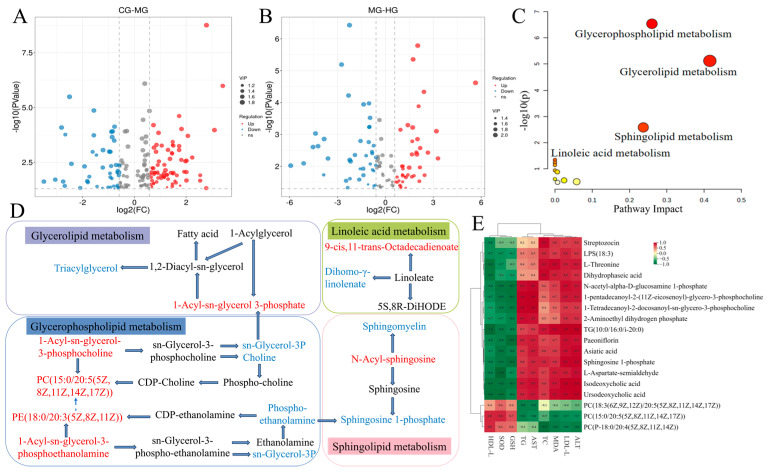
(**A**) Volcano map of the CG and MG. (**B**) Volcano map of the MG and HG. (**C**) Bubble plots. (**D**) Diagram of DMs involved in metabolic pathways. (**E**) Correlation analysis of DMs with biochemical indicators. Red indicates that metabolites were up-regulated, and blue indicates that metabolites were down-regulated in subfigure (**D**).

We further utilized OPLS-DA to investigate the differences in metabolites between the groups in mice. Overall, there was a clear separation between samples in the MG-CG, MG-PG, MG-LG, and MG-HG. The results of the permutation test for the OPLS-DA model indicated that R2 Xand Q2 were close to 1, which supported the validity and robustness of the model. The data obtained implied that HFD caused abnormal metabolic activities in mice while PAE intervention significantly modulated the substance metabolism in HFD mice. Metabolites with significant differences (DMs) were filtrated based on *t*-test *p* < 0.05 and VIP > 1, as previously describe [5]. These DMs were visualized using volcano plots (Figure 5A,B) and it was found that 173 DMs were screened in MG-CG, of which 105 were revised upwards and 68 downwards. Meanwhile, 109 DMs were identified in the HG-MG, of which 49 were up-regulated and 60 were down-regulated. Moreover, there were 50 DMs that occurred together in the CG-MG and HG-MG. Notably, 16 endogenous DMs were significantly reversed after PAE intervention (Table 2). Overall, PAE may alleviate NALFD-induced adverse effects by restoring the disrupted endogenous metabolic profile.

MetaboAnalyst5.0 was used to analyze 16 DMs with reversal phenomena and these important metabolic pathways involved in DMs were plotted as bubble diagrams in Figure 5C. The results revealed that the main metabolic pathways in which significant changes were monitored after intervention with PAE in HFD-induced NAFLD included glycerophospholipid metabolism, glycerolipid metabolism, sphingolipid metabolism, and linoleic acid metabolism. Subsequently, to reveal the potential relationships between DMs, we mapped metabolic networks (Figure 5D). The result revealed that the metabolic pathway of HFD-induced NAFLD was significantly changed after PAE treatment, especially glycerophospholipid metabolism. PAE treatment significantly increased the contents of PC (15:0/20:5 (5Z,8Z,11Z,14Z,17Z)), 1-Acyl-sn-glycero-3-phosphocholine, PE (18:0/20:3 (5Z,8Z,11Z)), and 1-Acyl-sn-glycero-3-phosphoethanolamine, while the contents of choline, sn-glycerol-3P, and phosphoethanolamine were significantly reduced. PC is the predominant phospholipid in mammalian cell membranes and subcellular organelles, comprising 45–55% of total phospholipid content, with PE following at 15–25% [43]. Approximately 70% of hepatic PC synthesis occurs through the CDP-choline pathway, with the remaining 30% from the phosphatidylethanolamine N-methyltransferase (PEMT) pathway [44]. As an important component of very low-density lipoprotein (VLDL) synthesis, PC is essential for the packaging and export of VLDL. The latter is the major lipoprotein synthesized and secreted by the liver, which is primarily responsible for the transport of synthesized TG to other tissues in the body. Thus, VLDL synthesis and transport will be inhibited when PC synthesis is impaired, leading to intrahepatic lipid accumulation, which promotes the development of NAFLD [45]. This was also consistent with our study described above that HFD-induced intracellular lipid accumulation and abnormally elevated lipid levels in NAFLD mice. PAE intervention resulted in a significant increase in liver levels of PC and PE, and alleviated the abnormal glycerophospholipid metabolism, which corresponded to the significant reduction in TC, TG, and LDL-C and enhancements in hepatic impairment by PAE in HFD mice.

Sphingolipid levels influence the progress of NAFLD through various mechanisms, including overweight, inflammation, insulin resistance, and oxidative stress [46]. Our study found that N-Acyl-sphingosine, which is important in sphingolipid metabolism, increased significantly after PAE intervention while Sph-1-phosphate (S1P) and sphingomyelin was down-regulated. N-Acyl-sphingosine is converted to dihydroceramide by ceramide synthase (CerS). Subsequently, ceramides can be converted into the bioactive signaling molecule called sphingosine (Sph) by neutral ceramidase (CDase), which can eventually be phosphorylated by Sph kinase (SphK) to S1P. S1P, a key metabolic intermediate and reactive lipid molecule, plays an essential position in the sphingolipid–glycerophospholipid metabolic pathway [47]. Research has shown that S1P promotes liver cell survival and anti-apoptosis, especially when the liver is subjected to oxidative stress or injury [48]. However, the up-regulation of N-Acyl-sphingosine expression did not promote SIP expression, possibly because the balance of different sphingolipids was still affected by multiple pathways and mechanisms. Therefore, further studies were still needed to reveal that the N-Acyl-sphingosine upstream of S1P was highly expressed while the expression of SIP was suppressed. All in all, these findings indicated that PAE has a beneficial impact on mitigating NAFLD, and involved the regulation of multiple metabolic pathways, especially those associated with lipid metabolism.

### 3.9. Potential Relationships Between Differential Metabolites and Biochemical Indices

The correlations of the DMs that showed significant reversal after PAE intervention and the main serum biochemical indices are shown in Figure 5E. Among them, L-threonine, paeoniflorin, L-aspartate-semialdehyde, 1-tetradecanoyl-2-docosanoyl-sn-glycero-3-phosphocholine, TG (10:0/16:0/i-20:0), isodeoxycholic acid, asiatic acid, dihydrophaseic acid, ursodeoxycholic acid, N-acetyl-alpha-D-glucosamine 1-phosphate, 2-aminoethyl dihydrogen phosphate, 1-pentadecanoyl-2-(11Z-eicosenoyl)-glycero-3-phosphocholine, and LPS (18:3) were strongly positively correlated with ALT, LDL-C, MDA, TC, AST, and TG and negatively correlated with GSH, SOD, and HDL-C. PC (15:0/20:5(5Z,8Z,11Z,14Z,17Z)), PC (18:3(6Z,9Z,12Z)/20:5(5Z,8Z,11Z,14Z,17Z)), and PC (P-18:0/20:4(5Z,8Z,11Z,14Z)) with ALT, LDL-C, MDA, TC, AST, and TG were negatively correlated, and strongly correlated with GSH, SOD, and HDL-C. The results suggested that significantly altered DMs after PAE intervention may alleviate HFD-induced NAFLD by affecting serum lipid profile and liver function.

### 3.10. PAE Suppresses Hepatic Lipid Accumulation via Regulating the AMPK/AKT/mTOR Pathway

**Figure 6:**
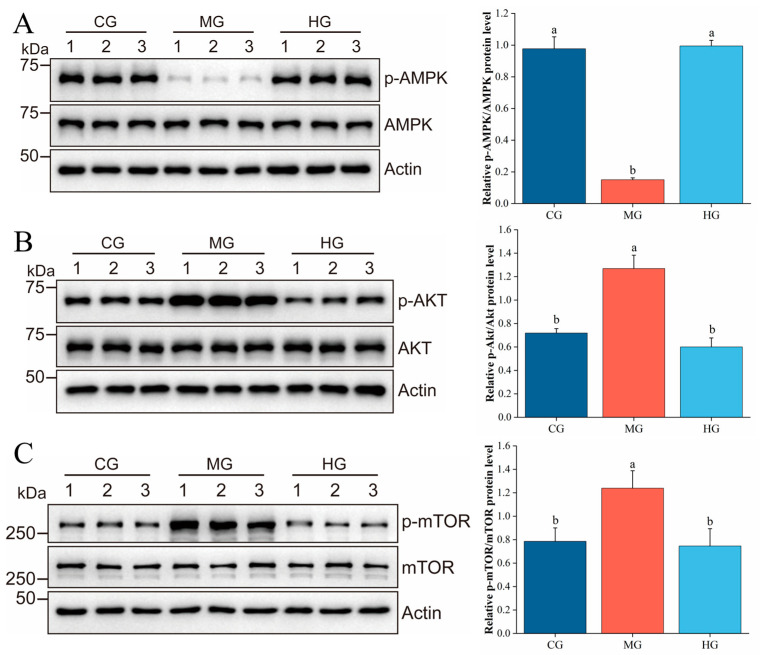
Effects of PAE on the expression of signaling protein in NAFLD with HFD. (**A**–**C**) Relative expression of p-AMPK/AMPK, p-AKT/AKT, and p-mTOR/mTOR. Different letters indicate significant differences between groups (*p* < 0.05).

It was clear that the occurrence and progression of NAFLD were caused by disorders in lipid synthesis and metabolism based on previous report combined with our current findings [49]. Notably, AMPK, mTOR, and protein kinase B (AKT) are critical modulators of glucose and lipid metabolism in the liver [50]. AMPK is essential for maintaining cellular energy balance and can alleviate NAFLD by influencing various signaling pathways related to lipid metabolism, autophagy, oxidative stress, inflammation, and insulin resistance. mTOR acts as a principal inhibitor of autophagy and plays a vital regulatory part in its initiation and progression [51]. When AMPK, functioning as an upstream regulator, experiences increased phosphorylation, it can effectively reduce mTOR levels [52], thereby enhancing autophagy. Autophagy is closely linked to fatty liver as intracellular lipid droplets become encapsulated by double-membrane autophagosomes before being delivered to lysosomes, where they are degraded into free fatty acids, contributing to the alleviation steatosis [53]. Additionally, autophagy not only modulates lipid metabolism and enhances insulin sensitivity but it also decreases oxidative stress and protects hepatocytes by mitigating cellular damage. Therefore, we monitored the exposure of relevant proteins in the AMPK/AKT/mTOR signaling pathway using WB, and the result is plotted in Figure 6. In this study, HFD induction was found to significantly regulate the AMPK/AKT/mTOR signaling pathway, in which AMPK phosphorylation was significantly down-regulated while mTOR and Akt phosphorylation were significantly up-regulated, consistent with a previous report [54]. After PAE intervention, AMPK phosphorylation was significantly up-regulated, while mTOR and Akt phosphorylation were significantly reduced. There was evidence to support that AMPK was activated to inhibit mTOR in the energy-deficient state, thereby reducing anabolism, enhancing autophagy, and promoting fatty acid oxidation and glucose utilization. Akt was activated to enhance anabolism and promote adipogenesis in the energy-rich state [55]. This was also consistent with the results from the above results, in which PAE intervention induced a decline in TC and TG levels accompanied by a remarkable reduction in lipid droplets observed through ORO staining. Overall, the current findings indicate that PAE intervention may reduce liver lipid deposition in NAFLD, which is hypothesized to be achieved by improving autophagy through the AMPK/AKT/mTOR signaling pathway.

### 3.11. The Impact of PAE on Gut Microbiota Dysbiosis in NAFLD Mice

**Figure 7:**
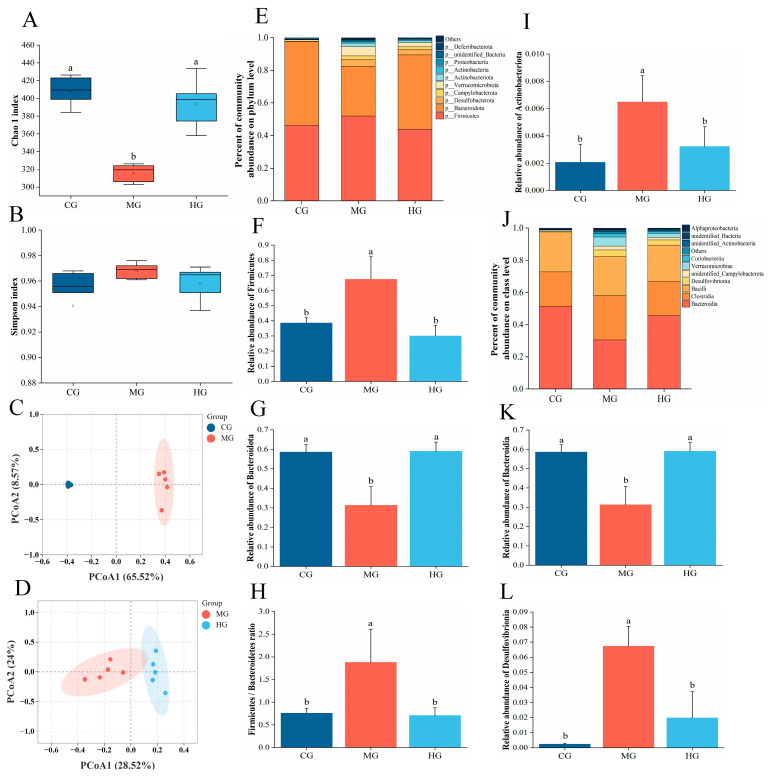
Effect of PAE on the gut microbiota of HFD mice. (**A**,**B**) α-diversity analysis (Chao 1 and Simpson index). (**C**,**D**) β-diversity analysis (PCA analysis). (**E**) Abundance analysis of phylum (the top 10 phylum). (**F**–**I**) Relative abundance of *Firmicutes*, *Bacteroidetes*, *Firmicutes*/*Bacteroidetes,* and *Actinobacteria*. (**J**) Abundance analysis of class, the top 10 genus. (**K**,**L**) Relative abundance of *Bacteroidia* and *Desulfovibrionia.* Different letters indicate significant differences between groups (*p* < 0.05).

NAFLD is often associated with alterations in gut microbiota, characterized by reduced microbial diversity and an increased prevalence of harmful bacterial strains [56]. Regulating the imbalance of gut microbiota offers new avenues for the prevention and treatment of NAFLD. Consequently, we employed 16S rDNA sequencing to investigate how PAE influences the gut microbiota of mice HFD. To evaluate gut microbial diversity and abundance, we utilized the α-diversity indices Chao1 and Simpson. Compared to the CG, the Chao1 and Shannon indices of the MG and HG showed varying degrees of change (Figure 7A,B), suggesting that both HFD and PAE intervention directly influenced the abundance of gut microbiota. Subsequently, the β-diversity of the microbial communities in each group was assessed using principal coordinates analysis (PCOA). It was found that the microbial communities of MG showed significant separation from those of CG and HG (Figure 7C,D), suggesting a substantial shift in the gut microbiome’s composition due to HFD feeding and PAE intervention. Afterwards, we analyzed the community composition and species abundance at both the phylum and class levels. At the phylum level, we noted a significant decrease in the relative abundance of *Bacteroidetes* in the MG than CG, while the relative abundance of *Firmicutes* was significantly increased (Figure 7F,G). After PAE intervention, the relative abundance of *Bacteroidetes* was markedly increased, and the proportion of *Firmicutes* declined markedly. Moreover, PAE treatment greatly reduced the *Firmicutes*/*Bacteroidetes* ratio (Figure 7H). It has been shown that the relative abundance of *Actinobacteria* often rises in NAFLD models [57], while the intervention with PAE can improve this trend. At the class level, the relative abundance of *Bacteroidia* was markedly decreased in MG and the relative abundance of *Desulfovibrionia* was markedly increased, while PAE intervention reversed the relative abundance of these. *Firmicutes* and *Bacteroidetes* are the two primary phyla of gut microbiota-influenced energy metabolism homeostasis [58]. A study has shown that chronic metabolic diseases often accompany chronic metabolic diseases, making the *Firmicutes*/*Bacteroidetes* ratio a crucial reference indicator for studying gut microbiota dysbiosis [59]. These were consistent with our findings that PAE improved homeostasis of the gut–liver axis by modulating the diversity and composition of the gut microbiota to alleviate NAFLD.

## 4. Conclusions

In summary, this study demonstrated that PAE could effectively alleviate HFD-induced liver injury in NAFLD mice mainly by attenuating insulin resistance, lipid accumulation, and oxidative stress. Furthermore, PAE replenishment significantly reversed abnormal liver lipid metabolic pathways and restored gut microbiota balance, while enhancing autophagy levels via regulating the AMPK/AKT/mTOR pathway, thereby preventing the progression of NAFLD. These findings underscore the potential of PAE as a functional component in the prevention or treatment of NAFLD and provide a theoretical basis for the utilization of phenolics in plants.

## Figures and Tables

**Table 1:** Top 20 polyphenols in PAE.

No.	TR (min)	Formula	Adduct	Mw (Da)	Compounds
1	3.88	C_9_H_12_O_2_	[2M+NH_4_]^+^	152.08	4-Ethyl-2-methoxyphenol
2	3.18	C_9_H_8_O_3_	[M−H]^−^	164.05	2-Hydroxycinnamic acid
3	4.57	C_27_H_30_O_15_	[M−H]^−^	594.16	Kaempferol 3-O-beta-D-glucopyranosyl-7-O-alpha-L-rhamnopyranoside
4	4.43	C_21_H_20_O_12_	[M−H]^−^	464.10	Hyperoside
5	4.69	C_25_H_24_O_12_	[2M−H]^−^	516.13	CID 153946
6	3.58	C_16_H_18_O_9_	[M−H]^−^	354.10	Chlorogenic Acid
7	4.28	C_27_H_30_O_14_	[M−H]^−^	578.16	Vitexin 2″-O-rhamnoside
8	3.74	C_26_H_28_O_14_	[2M−H]^−^	564.15	Neoschaftoside
9	4.83	C_32_H_22_O_10_	[M−H]^−^	566.12	Isoginkgetin
10	4.04	C_33_H_40_O_19_	[M−H]^−^	740.22	Kaempferol3-rhamninoside
11	3.26	C_27_H_30_O_16_	[M−H]^−^	610.15	Quercetin 3-(2-glucosylrhamnoside)
12	3.78	C_25_H_24_O_12_	[M−H_2_O−H]^−^	516.13	Isochlorogenic acid A
13	2.21	C_9_H_12_O_3_	[2M+NH_4_]^+^	168.08	(4-Hydroxy-3-methoxyphenyl) ethanol
14	3.78	C_17_H_14_O_5_	[2M−H]^−^	298.08	5,4′-DIMETHOXY-7-HYDROXYFLAVONE
15	3.52	C_27_H_32_O_15_	[M−H]^−^	596.17	Butrin
16	1.69	C_9_H_10_O_2_	[M+H]^+^	150.07	4′-Methoxyacetophenone
17	4.57	C_27_H_30_O_15_	[2M−H]^−^	594.16	3″-O-L-Rhamnopyranosylastragalin
18	4.49	C_10_H_10_O_4_	[M−H]^−^	194.06	Ferulic acid
19	3.58	C_9_H_8_O_4_	[M−H]^−^	180.04	Caffeic acid
20	6.32	C_23_H_27_NO_8_	[M−H]^−^	445.17	Narceine

**Table 2:** 16 endogenous DMs of apparently reversed.

No.	Metabolite	RT (min)	Molecular Formula	Pub Chem CID	MG vs. CG		HG vs. MG	
					FC ^a^	Trend	FC ^a^	Trend
1	PC (15:0/20:5 (5Z,8Z,11Z,14Z,17Z))	8.70	C_43_H_76_NO_8_P	52922332	0.66	↓	1.78	↑
2	PC (18:3(6Z,9Z,12Z)/20:5 (5Z,8Z,11Z,14Z,17Z))	7.37	C_46_H_76_NO_8_P	52922807	0.54	↓	4.42	↑
3	PC (P-18:0/20:4 (5Z,8Z,11Z,14Z))	7.51	C_46_H_84_NO_7_P	24779390	0.37	↓	1.97	↑
4	L-Threonine	0.82	C_4_H_9_NO_3_	6288	1.36	↑	0.89	↓
5	Paeoniflorin	2.82	C_23_H_28_O_11_	442534	2.17	↑	0.50	↓
6	L-Aspartate-semialdehyde	0.80	C_4_H_7_NO_3_	439235	1.09	↑	0.88	↓
7	1-Tetradecanoyl-2-docosanoyl-sn-glycero-3-phosphocholine	8.70	C_44_H_88_NO_8_P	24778638	1.66	↑	0.09	↓
8	TG (10:0/16:0/i-20:0)	6.58	C_49_H_94_O_6_	131777989	1.73	↑	0.71	↓
9	Isodeoxycholic acid	6.13	C_24_H_40_O_4_	164672	8.54	↑	0.20	↓
10	Asiatic acid	5.47	C_30_H_48_O_5_	119034	2.47	↑	0.39	↓
11	Dihydrophaseic acid	4.81	C_15_H_22_O_5_	11988272	3.54	↑	0.69	↓
12	Ursodeoxycholic acid	6.13	C_24_H_40_O_4_	31401	3.32	↑	0.49	↓
13	N-acetyl-alpha-D-glucosamine 1-phosphate	0.85	C_8_H_16_NO_9_P	440364	1.18	↑	0.80	↓
14	2-Aminoethyl dihydrogen phosphate	0.85	C_2_H_8_NO_4_P	1015	1.26	↑	0.82	↓
15	1-pentadecanoyl-2-(11Z-eicosenoyl)-glycero-3-phosphocholine	6.47	C_43_H_84_NO_8P_	52922324	2.27	↑	0.21	↓
16	LPS (18:3)	8.79	C_24_H_42_NO_9_P	52926285	2.40	↑	0.47	↓

↑: upregulated. ↓: downregulated. ^a^ Fold change equals the fold difference in concentration observed between two groups.

## Data Availability

The original contributions presented in this study are included in the article. Further inquiries can be directed to the corresponding authors.
